# Supplementary material for: The development and validation of a social media fatigue scale: From a cognitive-behavioral-emotional perspective
Source: PLoS One. 2021 Jan 22;16(1):e0245464. doi: 10.1371/journal.pone.0245464 (PMC7822276; doi:10.1371/journal.pone.0245464)
Supplement: S1 Appendix — (DOCX) [file pone.0245464.s001.docx]

**Supporting information**

**S1 Appendix. Social Media Fatigue Scale (SMFS)**

**Cognitive Experiences:**

1 I am frequently overwhelmed by the amount of information available on social media sites.

[1 我常常有被社交网络上的大量信息压垮的感觉。]

2 When searching for information on social media sites, I frequently just give up because there is too much to deal with.

[2 当我在社交网络上搜索信息时，我常常因为信息太多而放弃。]

3 I am likely to receive too much information when I am searching for something on social media sites.

[3 当我在社交网络上搜索时，我常常会接收到过多的信息。]

4 I usually avoid using social media for having received too much information.

[4 我会因社交网络推送了太多消息而回避它。]

5 I feel angry when I realize that social media has taken up too much of my time.

[5 当我意识到我被社交网络占据了过多时间的时候会感到生气。]

**Behavioral Experiences:**

6 I always have no idea what I am going to post on social media.

[6 我经常不知道要发布些什么消息。]

7 When I login a social media site, I’ll always forget whom I’ve intended to stalk on the site.

[7 我兴致勃勃地想去看某个人的消息，但是一打开社交网站我就忘了想找谁了。]

8 I’m likely to forget the content of the status which I have intended to repost.

[8 我在刷状态时看到了一条想转载的内容，但是过一会我就忘了。]

9 When I open a social media site, I may forget what I’ve intended to post on the social media site.

[9 我经常出现打开社交网站却忘了自己要发布什么的情况。]

10 It’s hard for me to come up with good ideas for updating status on social media sites.

[10 我有过在发状态时，不知道要发些什么的经历。]

**Emotional Experiences:**

11 I feel annoyed when I find there is too much unread information on social media sites.

[11 当我打开社交网络看到有很多未读消息时会感到生气。]

12 Functions in the social network (check-in, status updates, etc.) make me irritated.

[12 我会因为社交网络里的功能（签到、状态更新等）感到烦躁。]

13 I feel anxious when I was referred to (@) by others on the social media sites.

[13 当有人在社交网络里@我的时候我会感到焦虑。]

14 I feel nervous when receiving friend requests on social media sites.

[14 收到新好友请求的时候我会感到焦虑。]

15 Before I login in my social media account, I usually fear of receiving too much new messages.

[15 登录我的社交网络账户之前我会担心收到过多的新通知。]
