# Supplementary material for: The development and validation of a social media fatigue scale: From a cognitive-behavioral-emotional perspective
Source: PLoS One. 2021 Jan 22;16(1):e0245464. doi: 10.1371/journal.pone.0245464 (PMC7822276; doi:10.1371/journal.pone.0245464)
Supplement: Attachment — Submitted filename: Response to Reviewers.docx [file pone.0245464.s005.docx]

Dear editors and reviewers:

Thank you for your letter and the reviewers’ comments on our manuscript entitled *The development and validation of a social media fatigue scale: From a cognitive-behavioral-emotional perspective* (ID: PONE-D-20-08484). Those comments are very helpful for revising and improving our paper. We have studied the comments carefully and made corrections which we hope meet with approval. The main corrections are in the manuscript and the responds to the reviewers’ comments are as follows (the replies are marked in blue).

Replies to the reviewers’ comments:

Reviewer #1:

1. Abstract: The abstract is NOT adequately addressed study, it should re-structured according to the journal format.

Response: Thanks for your advice. We’ve re-structured a new abstract (line 22-45 in manuscript) according to your advice, and hope that it meets with approval.

2. Materials and methods:

Sampling technique: It has NOT been adequately described.

Recruitment of Subjects: It has NOT been Adequately described (Convenient sampling???)

Eligibility criteria; It has NOT been reported

Response: Thanks for your advice. We’ve added description of sampling technique (line 170-175 in manuscript), recruitment of subjects (line 170-175 in manuscript) and eligibility criteria (line 175-179 in manuscript). Hope that it will meet with approval.

3. Contribution to the literature: The authors should provide key points and the contribution of current study to literature and what messages are provided with the present study?

Response: Thanks for your advice. In order to provide key points and the contribution current study, we’ve revised the discussion part, and added a summary paragraph (line 387, line 401, line 412, and line 426-439 in manuscript)

4. Conclusions:

The authors reported that present study explored and verified the structure of SMF and the 15-item SMFS can be used to understand the phenomena of SMF for further study, which is NOT solid and conclusive at this stage.

Response: Thanks for your advice. We’ve changed the expression in both abstract (line 39-45 in manuscript) and discussion (line 426-439 in manuscript). Hope it will meet with approval.

5. However, the methods section is grossly deficient from epidemiological and statistical point view The authors recruited 30 participants offline for semi-structured interviews, and 1569 participants on SO JUMP (an online platform for data collection) It seems 30 subjects participants are non-randomly selected and too small sample size, which can be considered as a pilot study.

Response: Thanks for your advice. We’ve considered the semi-structured interviews as a pilot study and revised the relative part in the paper (line 154, line 170-172, line 249-269 in manuscript), besides, we added information on sampling technique (line 170-175 in manuscript), recruitment of subjects (line 170-175 in manuscript) and eligibility criteria (line 175-179 in manuscript). Hope that it will make the methods section more clarify.

Reviewer #2:

1. I recommend some changes and suggestions, they are: the exact repetition of writing should be avoided (see the sentence in the lines: 93 - 95 "when exposed to long-term excessive information, individuals with SMF will feel overloaded and eventually tend to refrain from using social media ", are also repeated on lines 100 - 101).

Response: Thanks for your advice! We’ve rewritten the latter sentence which can be seen in line 107-108 of the manuscript “long term exposure to excessive information makes individuals with SMF feel overloaded and ultimately tend to reduce their use of social media”.

2. I also suggest a revision in the sentence (lines 269-272) "However, based on the literature review and structure assumption for SMF, the factor loadings pattern for the current four-factor model was nearly the assumed structure for SMF and it was thus chosen as the optimal SMFS structure. " As described, it seems that the four-factor model was chosen, which is not true.

Response: Thank you for your advice, and we apologize for our carelessness! We’ve changed “four-factor model” into “three-factor model” (line 290 in the manuscript).

3. Still in the results, in Table 5 it is recommended to include the factorial loads of the items in all factors, highlighting them in each of the three dimensions. And put the acronym for standard deviation in capital letters.

Response: Thanks for your advice! We included the factorial loads of the items in all factors in Table 5, and we also put the acronym for standard deviation in capital letters.

4. Regarding reliability it is also recommended to include the value of McDonald's Omega.

Response: Thanks for your advice. We’ve calculated the McDonald’s Omega of the data, and replaced all the data of Cronbach’s alpha with McDonald’s Omega.

Once again, thank you very much for your constructive comments and suggestions which would help us both in English and in depth to improve the quality of the paper.

Kind regards,

Tao Xin

E-mail: [xtao_bnu@163.com](mailto:xtao_bnu@163.com)
